# Supplementary material for: The dynamics of Cryptococcus neoformans infection in Galleria mellonella
Source: mSphere. 2025 May 16;10(6):e00190-25. doi: 10.1128/msphere.00190-25 (PMC12188720; doi:10.1128/msphere.00190-25)
Supplement: Figure S1 — Time-lapse setup. [file msphere.00190-25-s0001.docx]

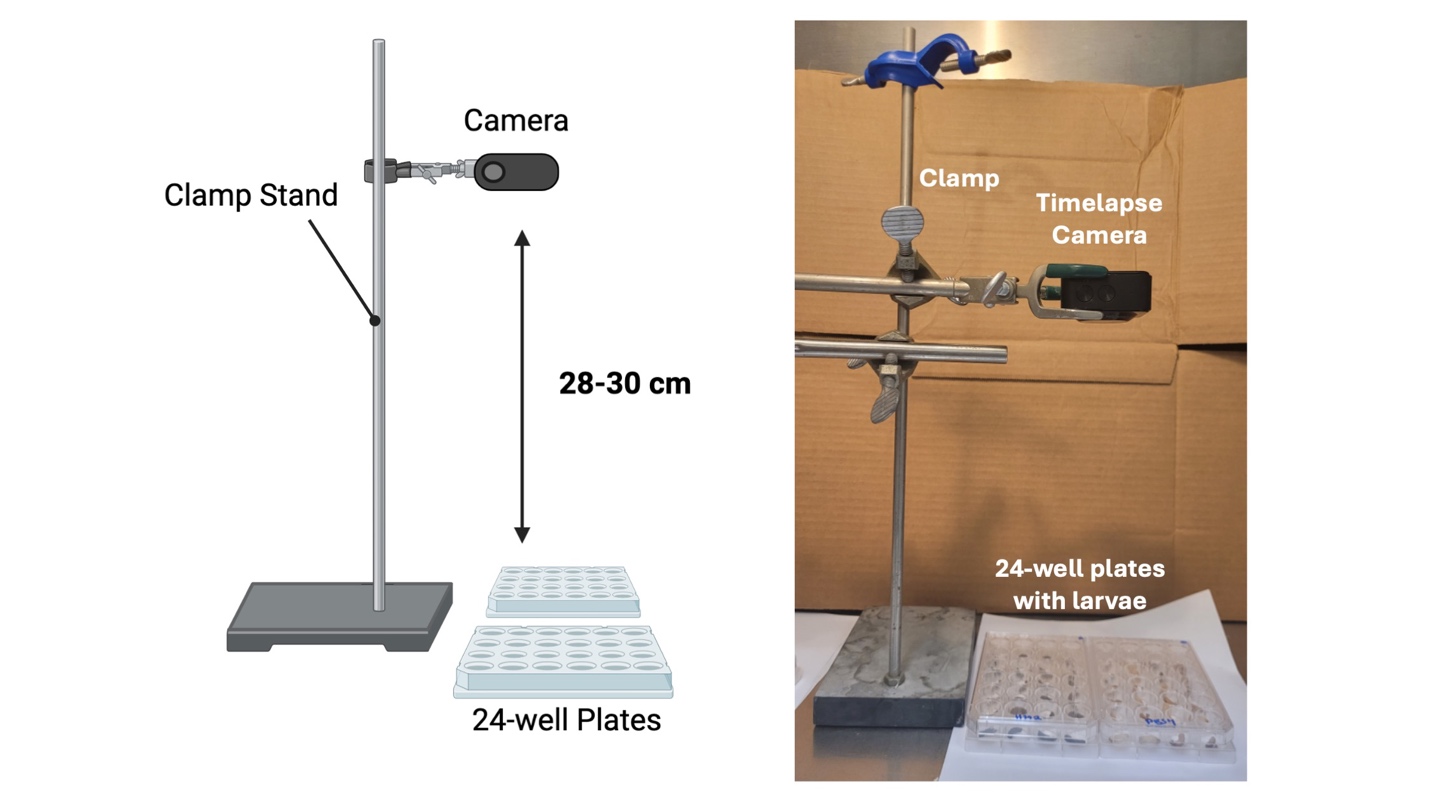


***Supplementary Figure 1. Timelapse* *photography set up.*** Timelapse camera is suspended above two 24-well plates containing *G. mellonella* larvae using a 3-pronged clamp attached to a support base.
